# The Effects of Fucoidan Dietary Supplementation on Growth Performance, Serum Antioxidant Capacity, Immune Function Indices and Intestinal Morphology in Weaned Kids

**DOI:** 10.3390/ani12050574

**Published:** 2022-02-24

**Authors:** Weiguang Yang, Jiayi Chen, Guangzhen Guo, Shengnan Wang, Su Peng, Zhenhua Gao, Zhihui Zhao, Ruixia Lan, Fuquan Yin

**Affiliations:** College of Coastal Agriculture Science, Guangdong Ocean University, Zhanjiang 524088, China; yangweiguang1@stu.gdou.edu.cn (W.Y.); chen17852729978@163.com (J.C.); guoguangzhen1@stu.gdou.edu.cn (G.G.); 2112004098@stu.gdou.edu.cn (S.W.); sugar7364@126.com (S.P.); xmsgzhh@126.com (Z.G.); zhzhao@gdou.stu.cn (Z.Z.)

**Keywords:** fucoidan, weaned kids, growth performance, antioxidant capacity, immune function

## Abstract

**Simple Summary:**

During the weaning period, the change of feed and separation from ewe induce weaning stress and may affect the growth and health of kids. The application of antibiotics could relieve weaning stress; however, their prophylactic application coerces researchers to find antibiotic alternatives to relieve weaning stress. Fucoidan is a natural plant extract widely used in animal production with antioxidant and immune-modulatory properties resulting in beneficial effects on the intestinal tract. In the present study, fucoidan dietary supplementation boosted antioxidant and immune functions, improved the morphology of the intestinal tract and promoted the growth performance of kids. These results indicated that fucoidan could be used to alleviate weaning stress in kids.

**Abstract:**

The purpose of this study was to evaluate the effects of fucoidan dietary supplementation on growth performance, organs’ relative weight, serum anti-oxidation markers, immune function indices and intestinal morphology in weaned kids. A total of 60 2-month-old weaned castrated male kids (Chuanzhong black goat) were used for this 30-day experiment and randomly allocated to four groups. The control group (CON) fed a basal diet, while the other three groups were provided with the same diet further supplemented with fucoidan at 0.1%, 0.3% or 0.5%, namely, F1, F2 and F3 groups, respectively. The results indicated that dietary fucoidan supplementation significantly increased (*p* < 0.05) the activity of catalase (CAT) when compared to the CON group on day 15. Moreover, the addition of fucoidan at 0.3% and 0.5% significantly increased (*p* < 0.05) the activities of glutathione peroxidase (GSH-Px) and total superoxide dismutase (T-SOD). On day 30, dietary fucoidan supplementation significantly reduced (*p* < 0.05) the feed conversion rate (FCR), contents of tumor necrosis-α (TNF-α), interleukin-1β (IL-1β) and interleukin-6 (IL-6), while it significantly increased (*p* < 0.05) the activity of total superoxide dismutase (T-SOD), the content of immunoglobulin G (IgG) and the villus height (VH) of the duodenum. Moreover, dietary 0.3% and 0.5% fucoidan supplementation significantly increased (*p* < 0.05) the villus height (VH) of the jejunum and ileum and significantly reduced (*p* < 0.05) the crypt depth (CD) of ileum. In conclusion, dietary fucoidan had positive effects on growth performance, serum anti-oxidation, immune function and intestinal morphology of weaned kids.

## 1. Introduction

In modern small ruminants’ production, weaning stress is an inevitable problem observed in small animals. Weaning stress had negative effects on antioxidant capacity, immunity, intestinal morphology and growth performance [1,2,3]. Formerly, antibiotics were widely used to alleviate weaning stress [4]. However, the ban of antibiotic use in feed worldwide forces researchers to find antibiotic alternatives. Fucoidan, a kind of macromolecular polysaccharide rich in sulfate, occurs in the cell walls of brown algae, mucous matrix and some marine invertebrates [5,6]. Fucoidan has been proven to possess many biological properties, including immunomodulatory, antioxidant and antibacterial properties, resulting in the promotion of animal growth [7,8,9,10]. Thus, fucoidan was widely used in functional foods and animal production. Dietary fucoidan administration increased feed intake, daily gain and feed efficiency and also had beneficial effects on intestinal morphology, antioxidant capacity and immune function in weaning pigs [11,12,13]. Similar results are also reported in chickens and fish [14,15,16]. In summary, fucoidan could be used as an environmentally friendly substitute for antibiotics in diets to improve growth in pigs, chicken and fish. However, data on the application of fucoidan in small ruminants are scarce. Therefore, the purpose of this study was to evaluate the effects of fucoidan on growth performance, organs’ relative weight, antioxidant markers, immunity indices and intestine morphology in weaned kids.

## 2. Materials and Methods

### 2.1. Animals, Diet and Experimental Design

The experimental protocol applied in this study followed the guidelines of the Animal Care and Use Committee of Guangdong Ocean University.

The fucoidan used in this study was provided by a company (Mingyue Hailin Fucoidan Biotechnology Co., Ltd., Qingdao, China). Fucoidan had the form of a powder with a yellow color and a smell of seaweed. The purity of fucoidan was 98%, and the sulfate ion content was 28.9%.

A total of 60 two-month-old weaned castrated male kids (Chuanzhong black goat) with an average initial body weight of 12.5 ± 0.5 kg were used in this 30-day experiment. Kids were weaned at 60 days and randomly allocated to 4 treatments with 15 replications. The control group (CON) was fed with a basal diet, while the other three treatment groups were fed with the same diet further supplemented with fucoidan at 0.1% (F1 group), 0.3% (F2 group) and 0.5% (F3 group). Three kids were placed in a pen (each pen = 3.1 m × 2.5 m × 1 m). The basal diet (Table 1) was formulated to meet or exceed the nutrient requirement of the Feeding standard of Goat, China (NY/T 861-2004).

Before the trial started, the sheep house was cleaned and sterilized. A 7-day pre-trial was conducted first, during which vaccination, deworming and numbering were performed. The roughage consisted of silage and *Aneurolepidium* Chinese hay. The kids were fed twice a day at 8:30 am and 17:30 pm, with access to clean drinking water available ad libitum. Fucoidan was manually mixed into the concentrate. The kids were fed concentrate first and then roughage.

### 2.2. Growth Performance

Body weight was determined on day 1, 15 and 30, the feed intake was recorded daily. The average daily gain (ADG), average daily feed intake (ADFI) and feed conversion rate (FCR) were also calculated.

### 2.3. Sample Collection and Organs’ Relative Weight

On days 15 and 30, blood samples were collected from the jugular vein, and then they were centrifuged at 3500 g for 10 min (4 °C). The serum was collected and stored at −20 °C for later analysis.

Kids were fasted for 12 h prior to slaughter at the end of the trial. Six kids of similar weight per group were selected for slaughter. About 2.5 cm segment of the duodenum, jejunum and ileum were trimmed and used for morphological indices. Samples of tissue were washed with PBS, then fixed in paraformaldehyde for histological evaluations. Finally, organs were weighed.

The organ relative weight was calculated by the following formula: organ index (%) = organ weight/body weight × 100%.

### 2.4. Serum Antioxidant

The activities of total antioxidant capacity (T-AOC), total superoxide dismutase (T-SOD), catalase (CAT), glutathione peroxidase (GSH-Px) and malondialdehyde (MDA) content were measured using commercial kits according to manufacturer’s guidelines (Nanjing Jiancheng Bioengineering Institute, Jiangsu, China).

### 2.5. Serum Immunity

The contents of immunoglobulin G (IgG), interleukin-1β (IL-1β), interleukin-6 (IL-6), interleukin-2 (IL-2), interleukin-10 (IL-10) and tumor necrosis factor-α (TNF-α) were estimated by enzyme-linked immunosorbent assay kits according to manufacturer’s guidelines (Jiangsu Meimian industrial Co., Ltd., Jiangsu, China).

### 2.6. Intestinal Histomorphology

The samples from the duodenum, jejunum and ileum were fixed in paraformaldehyde for 24 h at room temperature and subsequently dehydrated through a graded ethanol series, cleared with xylene and embedded in paraffin. Then, tissues were cut into 5 μm-thick continuous sections. Finally, the sections were stained with hematoxylin for 2 min and eosin for 40 s, and then dehydrated and mounted on slides. The morphological parameters were measured by Image Pro Plus 6.0 software (Media Cybernetics, Silver Spring, MD). The morphological parameters of the intestinal tract included villus height (VH), crypt depth (CD) and radio of villus height to crypt depth (VCR).

### 2.7. Statistical Analysis

All statistical analyses were performed using SPSS 26.0 via one-way ANOVA, and differences were detected by Ducan’s multi-range test. The results are expressed as mean ± standard error of the mean (SEM), and differences are considered significant at *p* < 0.05.

## 3. Results

### 3.1. Grouth Performance

As shown in Table 2, kids fed F2 and F3 diets had a higher final body weight than those fed the CON and F1 diets. Kids fed diets with fucoidan significantly reduced (*p* < 0.05) FCR compared to those fed the CON diet during days 16 to 30 and the overall period. Kids fed F2 and F3 diets had significantly higher (*p* < 0.05) values for ADG and ADFI than those fed the CON diet during days 16 to 30 and the overall period. No significant differences were observed for growth performance among treatments during days 1 to 15.

### 3.2. Organs’ Relative Weight

As shown in Table 3, no significant differences were observed for organs’ relative weight among the experimental groups.

### 3.3. Serum Antioxidant Capacity

**Figure 1:**
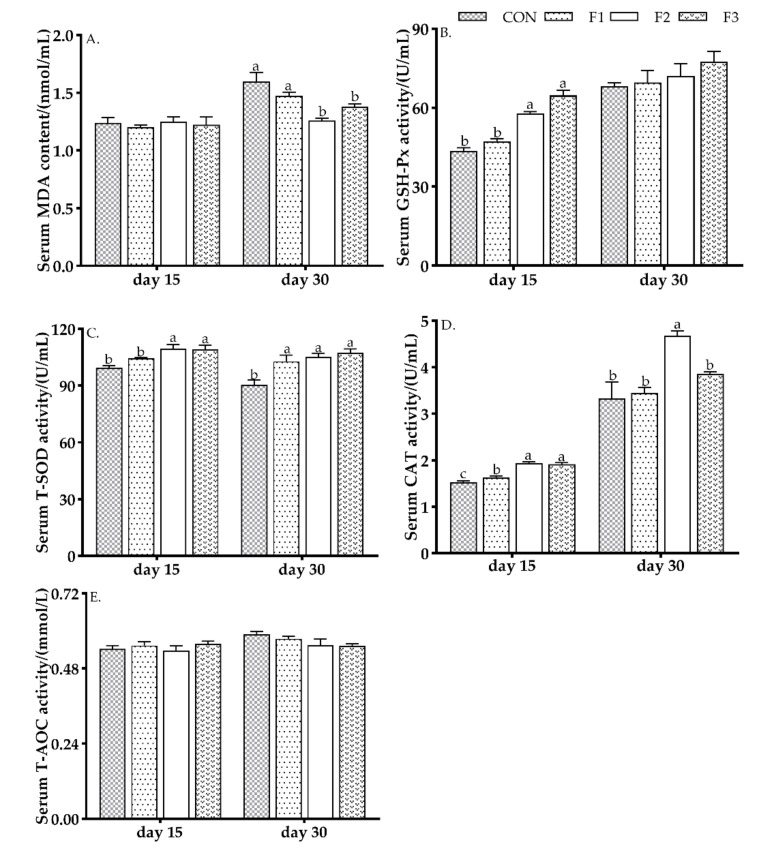
Effects of dietary fucoidan administration on antioxidant indexes in weaned kids. (**A**) serum MDA content; (**B**) serum GSH-Px activity; (**C**) serum T-SOD activity; (**D**) serum CAT activity; (**E**) serum T-AOC activity. Results are presented as mean ± SEM (*n* = 6). ^a, b, c^ means significantly different (*p* < 0.05). CON, basal diet; F1, basal diet +0.1% fucoidan; F2, basal diet +0.3% fucoidan; F3, basal diet +0.5% fucoidan. MDA, malondialdehyde; GSH-Px, glutathione peroxidase; T-SOD, total superoxide dismutase; CAT, catalase; T-AOC, total antioxidant capacity.

As shown in Figure 1, on day 15, kids fed diets with fucoidan had a significantly increased CAT activity (*p* < 0.05). Lambs fed F2 and F3 diets showed significantly increased (*p* < 0.05) activity of GSH-Px and T-SOD than those kids fed CON and F1 diets. No significant differences were observed for MDA and T-AOC among treatments.

On day 30, kids fed diets with fucoidan showed significantly increased (*p* < 0.05) activity of T-SOD. Kids fed F2 and F3 diets had a significantly reduced (*p* < 0.05) MDA content than those fed CON and F1 diets. Kids fed F2 diet had a significantly increased (*p* < 0.05) activity of CAT than those kids fed CON, F1 and F2 diets. No significant differences were observed for GSH-Px and T-AOC content among treatments.

### 3.4. Serum Immuntiy Indices

**Figure 2:**
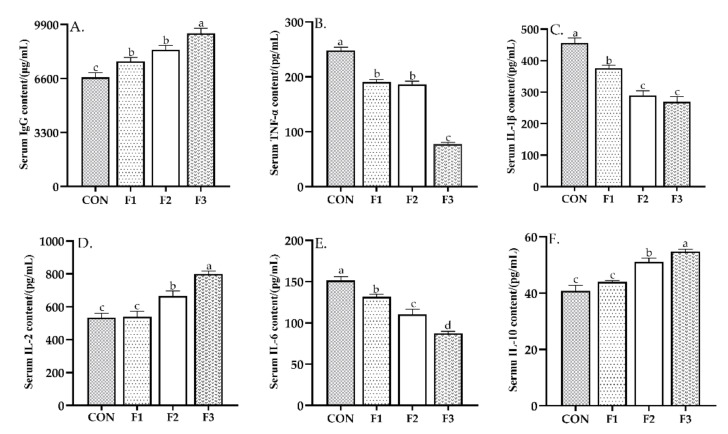
Effects of dietary fucoidan administration for 30 days on serum immunity indexes in weaned kids. (**A**) serum IgG content; (**B**) serum TNF-α content; (**C**) serum IL-1β content; (**D**) serum IL-2 content; (**E**) serum IL-6 content; (**F**) serum IL-10 content. Results are presented as mean ± SEM (*n* = 6). ^a–d^ means significantly different (*p* < 0.05). CON, basal diet; F1, basal diet +0.1% fucoidan; F2, basal diet +0.3% fucoidan; F3, basal diet +0.5% fucoidan. IgG, Immunoglobulin G; TNF-α, tumor necrosis factor-α; IL-1β, interleukin-1β; IL-2, interleukin-2; IL-6, interleukin-6; IL-10, interleukin-10.

As shown in Figure 2, kids fed diets with fucoidan had significantly higher IgG content than that fed with CON diet on day 30 (*p* < 0.05). Kids fed diets with fucoidan had significantly lower TNF-α, IL-1β and IL-6 contents than those fed CON diet (*p* < 0.05). On the other hand, F2 and F3 animals had significantly higher IL-2 and IL-10 contents than those fed CON and F1 diets (*p* < 0.05).

### 3.5. Intestinal Morphology

**Figure 3:**
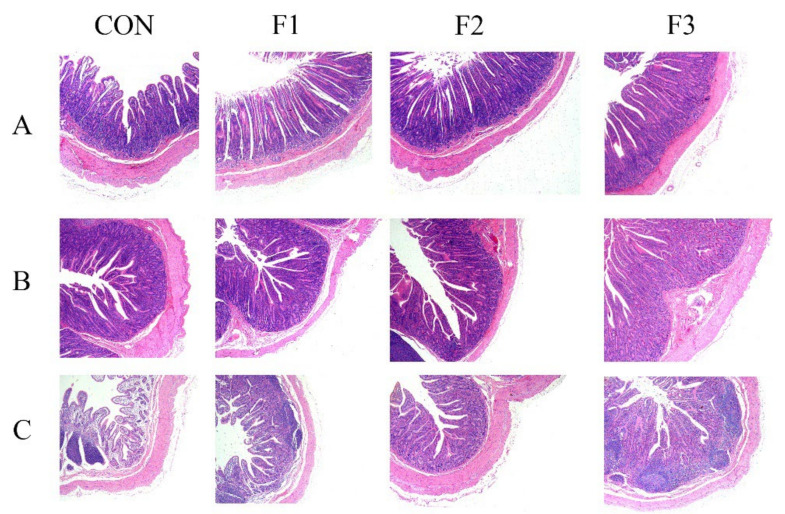
Micrograph of duodenum, jejunum and ileum supplemented with fucoidan in the diet of weaned kids: (**A**) duodenum; (**B**) jejunum; (**C**) ileum. F1, basal diet +0.1% fucoidan; F2, basal +0.3% fucoidan; F3, basal +0.5% fucoidan.

The effects of dietary fucoidan administration on intestinal morphology are illustrated in Figure 3. From the HE staining, we could see that the intestinal villi were denser and longer than the CON in the duodenum, jejunum and ileum. As shown in Table 4, kids fed with fucoidan-supplemented diets had significantly higher VH in the duodenum on day 30 (*p* < 0.05). Moreover, kids fed with F2 and F3 diets significantly increased (*p* < 0.05) the VCR in the duodenum and ileum than those fed with CON and F1 diets. Kids fed with F2 and F3 diets had significantly lower (*p* < 0.05) CD values in the ileum than those fed with CON and F1 diets. Kids fed with F2 and F3 diets significantly increased (*p* < 0.05) the VH in the ileum than those fed with CON and F1 diets. However, no significant differences were observed for CD in the duodenum and CD or VCR in the jejunum among treatments.

## 4. Discussion

### 4.1. Growth Performance

In general, weaning stress adversely affects the growth performance of kids by reducing feed intake, feed efficiency, immune suppression and increasing intestinal damage [17,18]. In our study, dietary fucoidan administration had no significant effects on growth performance among treatments during days 1 to 15. In support of our results, Rattigan et al. [19] reported that dietary fucoidan supplementation had no significant effects on feed intake and daily gain during days 1 to 14; however, supplemented weaner pigs showed higher feed intake and ADG than those in the CON group during days 16 to 30 and 1 to 30. Similarly, Draper et al. [20] indicated that dietary fucoidan administration increased feed intake and decreased FCR in weaning pigs. We speculated that the improved effects of fucoidan on the growth performance in this study might be related to the improvement of feed intake, antioxidant capacity, immune function and intestinal structure.

### 4.2. Serum Antioxidant

Weaning can lead to the excessive production of oxygen-free radicals, which results in oxidative stress and is linked with reduced growth performance [21,22]. SOD, GSH-Px and CAT activities are the first lines against oxidative injury, and MDA was the final product of lipid peroxidation [23,24]. In this study, dietary fucoidan administration increased the activities of GSH-Px, SOD and CAT on day 15 and decreased the content of MDA on day 30. Similarly, Yang et al. [25] reported that dietary supplementation with 0.1% fucoidan increased the activities of CAT and SOD and decreased the content of MDA in *Pelteobagrus fulvidraco*. Zhang et al. [26] indicated that supplementation with fucoidan improved the activities of SOD, CAT and GSH and reduced the content of MDA in drosophila geriatric. In summary, the improvement of antioxidant capacity with dietary fucoidan supplementation might be related to the increased activities of antioxidant enzymes and the reduced lipid oxidation values.

### 4.3. Serum Immunity

Previous research confirmed that weaning reduced immunity function, making invasion by external pathogenic microorganisms easier [27]. Immunoglobulin and cytokines are an important part of the immune system [28]. The immunoglobulins protect the body by removing pathogenic microorganisms and harmful molecules [29]. Cytokines play a key role in inflammation and anti-inflammatory processes [30]. In this study, dietary fucoidan administration increased the content of IgG, IL-2 and IL-10 in serum while reducing the content of IL-1β, TNF-α and IL-6. In agreement with our study, Lean et al. [31] reported that dietary fucoidan administration reduced the content of IL-1β in colons in mice with colitis. Tomori et al. [32] indicated that fucoidan increased the contents of IgG and IL-2 in serum by promoting the proliferation of immune cells and reducing the contents of IL-4 and IL-5 in spleens of mice. Moreover, Delma et al. [33] indicated that fucoidan exerted anti-inflammatory activity by regulating the NF-κB signaling pathway and reducing the expression of p53. Taken together, the improvement of immunological functions with dietary fucoidan supplementation may be related to the regulation of inflammatory-related factors release.

### 4.4. Intestinal Morphology

At weaning, the reduced villus height and increased crypt depth are related to stress [34,35]. In this study, dietary fucoidan administration increased the villus height of the duodenum, jejunum and ileum and decreased the crypt depth of the ileum. Our results are in line with that of Leonard et al. [36], who reported that dietary fucoidan administration in weaning pigs increases villus height and VCR of jejunum. Similarly, Walsh et al. [12] indicated that dietary fucoidan supplementation increased the villus height of small intestinal in post-weaning pigs. According to current studies, it indicated that dietary fucoidan administration could improve the intestinal morphology of the small intestine.

## 5. Conclusions

In summary, this study showed that fucoidan dietary supplementation improved the feed intake, daily gain, antioxidant capacity, immune status and intestinal morphology in weaned kids.

## Figures and Tables

**Table 1:** The composition and level of basal diet (air-dry basis).

Items	Content
Ingredients (%)	
Pennisetum purpureum	35.00
Corn	40.89
Soybean meal	13.98
Wheat bran	7.15
NaCl	0.65
CaHPO_4_	0.84
Limestone	0.84
Premix ^1^	0.65
Total	100
Nutrient level	
DM (%)	88.86
ME ^2^ (MJ/Kg)	10.43
CP (%)	12.06
NDF (%)	30.06
ADF (%)	16.39
Ca (%)	0.76
P (%)	0.54

^1^ The premix provided the following per kg of diets: VA 8 000 IU, VD 2 000 IU, VE 40 IU, Cu 12 mg, Fe 70 mg, Mn 50 mg, Zn 80 mg, I 1.0 mg, Se 0.27 mg and Co 0.3 mg. ^2^ ME was a calculated value.

**Table 2:** Effects of fucoidan administration on growth performance in weaned kids.

Item ^1^	CON	F1	F2	F3	SEM ^2^	*p*-Value
BW of day 1 (kg)	12.20	11.38	12.27	12.98	0.99	0.26
BW of day 15 (kg)	12.47	11.53	13.25	12.78	1.08	0.12
BW of day 30 (kg)	12.85 ^b^	12.88 ^b^	15.28 ^a^	15.13 ^a^	1.09	0.02
Day 1 to 15						
ADG (g/d)	30.00	31.78	51.78	53.22	10.41	0.10
ADFI (g/d)	432.01	427.02	484.05	445.01	24.41	0.16
FCR (g)	14.52	12.01	9.41	11.62	1.61	0.08
Day 16 to 30						
ADG (g/d)	80.01 ^a^	110.01 ^a^	141.78 ^b^	121.04 ^b^	13.90	0.01
ADFI (g/d)	569.05 ^a^	620.67 ^b^	711.03 ^c^	863.04 ^d^	16.08	<0.01
FCR (g)	9.97 ^b^	5.79 ^a^	5.02 ^a^	6.22 ^a^	0.69	<0.01
Overall						
ADG (g/d)	45.00 ^a^	50.06 ^a^	100.33 ^c^	72.01 ^b^	9.71	0.02
ADFI (g/d)	501.09 ^a^	525.08 ^a^	597.01 ^b^	565.02 ^b^	22.71	0.01
FCR (g)	11.25 ^c^	10.60 ^b^	6.05 ^a^	8.25 ^a^	1.08	0.01

^1^ CON, basal diet; F1, basal diet +0.1% fucoidan; F2, basal diet +0.3% fucoidan; F3, basal diet +0.5% fucoidan. BW, body weight; ADG, average daily gain; ADFI, average daily feed intake; FCR, feed conversion rate. ^2^ SEM, standard error of the mean. ^a–d^ Values in the same row with different letters are significantly different (*p* < 0.05). Results are presented as mean ± SEM (*n* = 6).

**Table 3:** Effects of fucoidan administration on organ index in weaned kids.

Item ^1^, %	CON	F1	F2	F3	SEM ^2^	*p*-Value
Heart index	0.41	0.40	0.39	0.40	0.50	0.13
Liver index	1.56	1.85	1.52	1.65	0.14	0.15
Spleen index	0.14	0.15	0.15	0.13	0.01	0.22
Lung index	1.15	1.37	1.170	1.37	0.20	0.61
Kidney index	0.33	0.35	0.33	0.33	0.03	0.91
Small intestine index	4.32	4.10	4.21	4.21	0.30	0.06

^1^ CON, basal diet; F1, basal diet +0.1% fucoidan; F2, basal diet +0.3% fucoidan; F3, basal diet +0.5% fucoidan. ^2^ SEM, standard error of mean.

**Table 4:** Effects of dietary fucoidan administration on intestinal morphology in weaned kids.

Item ^1^	CON	F1	F2	F3	SEM ^2^	*p*-Value
Duodenum						
VH (μm)	514.83 ^c^	657.89 ^b^	841.39 ^a^	904.28 ^a^	48.38	<0.01
CD (μm)	420.44	358.94	407.33	244.56	68.56	0.07
VCR	1.42 ^b^	1.94 ^b^	2.21 ^b^	3.02 ^a^	0.51	<0.01
Jejunum						
VH (μm)	578.50 ^b^	657.06 ^b^	708.28 ^a^	774.39 ^a^	43.41	<0.01
CD (μm)	345.11	337.67	412.45	467.00	65.75	0.19
VCR	1.70	2.15	1.94	1.75	0.36	0.59
Ileum						
VH (μm)	578.50 ^b^	657.06 ^b^	708.28 ^a^	771.50 ^a^	44.05	<0.01
CD (μm)	420.45 ^a^	435.61 ^a^	314.22 ^b^	354.00 ^b^	85.80	0.04
VCR	1.87 ^b^	1.76 ^b^	2.44 ^b^	3.82 ^a^	0.58	0.02

^1^ CON, basal diet; F1, basal diet +0.1% fucoidan; F2, basal diet +0.3% fucoidan; F3, basal diet +0.5% fucoidan. VH, villus height; CD, crypt depth; VCR, ratio of villus height to crypt depth. ^2^ SEM, standard error of mean. ^a–c^ Values in the same row with different letters are significantly different (*p* < 0.05). Results are presented as mean ± SEM (*n* = 6).

## Data Availability

The data presented in this study are available on request from the corresponding author.
